# The diabetic ketoacidosis

**DOI:** 10.1186/1824-7288-40-S1-A58

**Published:** 2014-08-11

**Authors:** Francesca Cardella

**Affiliations:** 1 Pediatrics Diabetology Unit Children Hospital “G. Di Cristina”, Department of Pediatrics, University of Palermo, Palermo, Italy

## 

The Diabetic Ketoacidosis (DKA) is still today a medical emergency in pediatrics. Despite the latest great sensibilization of the population and the doctors, the risk of DKA has not yet been eliminated and this pathology is still occurring in 25 to 40% of diabetes onset cases, in already diagnosed patients with poor compliance (10%), in patients undergoing acute medical or surgical events or in patients in Continuous Subcoutaneous Insulin Therapy (CSII). In toddlers (0-3 years) it is twice more frequent than in the following ages and is characterized by the presence of more serious clinical dehydratation (>10%) and neurological signs (obnubilation 40%). The other category at risk is represented by teen-agers, who may suffer from DKA at diabetes onset (scarce vigilance or reticence on the problems), or in diabetes treatment when there is poor compliance . In affected patients, missed recognition can influence morbility and mortality rates. Despite the improvement in DKA management and therapy, a lot of controversies have been encountered in literature. For the insulin therapy a wide consent exists on the need to use small doses of regular insulin for continuous intravenous administration (0.05- 0.1U/Kg/h). For children hydratation the most recent recommendations are not to overcome 5-10 ml/Kg/h in the first two hours (max 250 ml/h: ISPAD-IDF 2011, ADA 2013) and to continue hydratation slowly calculating the body surface area so as not to exceed 3 lt/mq/day (average 2000-2500 ml/mq/day) . The careful controls of plasmatic electrolytes (opportune integrations particularly of potassium deficit: 20-40 mEq/lt, 50% of KCl + 50% of KPO4) and of glycemia are suggested (to avoid too rapid falls: when glycemia <250- 300 mg/dl replace the sol. NaCl 0.9% N with mixed sol. constituted by 50% of Glucose 10% sol. and 50% of NaCl 0.9% N sol.). The follow-up of clinical patient conditions and the EKG evaluation prevent rapid falls of kaliemia with well-known cardiac consequences .

The success of the treatment is nevertheless tightly connected to a correct management of rehydratation, of metabolic acidosis and of electrolyte deficit replacement more than on insulin therapy, aimed at avoiding the most dangerous complication of DKA: cerebral oedema, that seems to be more frequent in patients with more severe onsets, particularly in those with low paCO2 and high levels of urea nitrogen, but seems to be correlated also to the rapid administration of fluids and to the inadequate use of NaHCO3.

